# Myopathy With *SQSTM1* and *TIA1* Variants: Clinical and Pathological Features

**DOI:** 10.3389/fneur.2018.00147

**Published:** 2018-03-19

**Authors:** Zhiyv Niu, Carly Sabine Pontifex, Sarah Berini, Leslie E. Hamilton, Elie Naddaf, Eric Wieben, Ross A. Aleff, Kristina Martens, Angela Gruber, Andrew G. Engel, Gerald Pfeffer, Margherita Milone

**Affiliations:** ^1^Department of Laboratory Medicine and Pathology, Mayo Clinic, Rochester, MN, United States; ^2^Department of Clinical Genomics, Mayo Clinic, Rochester, MN, United States; ^3^Department of Clinical Neurosciences, University of Calgary, Calgary, AB, Canada; ^4^Hotchkiss Brain Institute, University of Calgary, Calgary, AB, Canada; ^5^Department of Neurology, Mayo Clinic, Rochester, MN, United States; ^6^Department of Pathology and Laboratory Medicine, University of Calgary, Calgary, AB, Canada; ^7^Department of Biochemistry and Molecular Biology, Mayo Clinic, Rochester, MN, United States; ^8^Prevention Genetics, Marshfield, WI, United States

**Keywords:** distal myopathy, myofibrillar myopathy, respiratory insufficiency, rimmed vacuoles, SQSTM1, TIA1

## Abstract

**Objective:**

The aim of this study is to identify the molecular defect of three unrelated individuals with late-onset predominant distal myopathy; to describe the spectrum of phenotype resulting from the contributing role of two variants in genes located on two different chromosomes; and to highlight the underappreciated complex forms of genetic myopathies.

**Patients and methods:**

Clinical and laboratory data of three unrelated probands with predominantly distal weakness manifesting in the sixth-seventh decade of life, and available affected and unaffected family members were reviewed. Next-generation sequencing panel, whole exome sequencing, and targeted analyses of family members were performed to elucidate the genetic etiology of the myopathy.

**Results:**

Genetic analyses detected two contributing variants located on different chromosomes in three unrelated probands: a heterozygous pathogenic mutation in *SQSTM1* (c.1175C>T, p.Pro392Leu) and a heterozygous variant in *TIA1* (c.1070A>G, p.Asn357Ser). The affected fraternal twin of one proband also carries both variants, while the unaffected family members harbor one or none. Two unrelated probands (family 1, II.3, and family 3, II.1) have a distal myopathy with rimmed vacuoles that manifested with index extensor weakness; the other proband (family 2, I.1) has myofibrillar myopathy manifesting with hypercapnic respiratory insufficiency and distal weakness.

**Conclusion:**

The findings indicate that all the affected individuals have a myopathy associated with both variants in *SQSTM1* and *TIA1*, respectively, suggesting that the two variants determine the phenotype and likely functionally interact. We speculate that the *TIA1* variant is a modifier of the *SQSTM1* mutation. We identify the combination of *SQSTM1* and *TIA1* variants as a novel genetic defect associated with myofibrillar myopathy and suggest to consider sequencing both genes in the molecular investigation of myopathy with rimmed vacuoles and myofibrillar myopathy although additional studies are needed to investigate the digenic nature of the disease.

## Introduction

T-cell intracellular antigen-I (*TIA1*, chr.2) is a broadly expressed RNA-binding protein required for the formation of cytoplasmic stress granules, which play a crucial role in preventing misfolded protein accumulation (1). To date, a single *TIA1* mutation (p.Glu384Lys) is known to cause a late-onset myopathy with rimmed vacuoles (MRVs) (2, 3). *TIA1* mutations were recently detected in amyotrophic lateral sclerosis (ALS)/frontotemporal dementia (FTD) (1), but a subsequent study suggested exerting caution on the causality of the *TIA1* variants in ALS (4).

Sequestosome-1 (*SQSTM1*, chr.5) is a scaffolding protein involved in multiple cellular processes, including apoptosis, cell survival, and autophagy. Its numerous domains allow SQSTM1 to serve as a frame for multiprotein complexes and regulator of ubiquitinated protein turnover (5). *SQSTM1* mutations have been linked with a spectrum of phenotypes, including Paget disease of bone (PDB), ALS, FTD, and MRV (5–7). Hence, *SQSTM1* mutations can lead to a multisystem proteinopathy although with incomplete penetrance. A single *SQSTM1* mutation (c.1165+1G>A) has been linked to MRV in one family and an unrelated patient (6). This patient was subsequently found to carry a coexisting *TIA1* variant (c.1070A>G, p.Asn357Ser) by Evila et al. (8). Evila et al.’s study reported also an additional sporadic MRV case carrying the same *TIA1* variant but a different *SQSTM1* mutation (p.Pro392Leu), which is known to cause PDB, ALS, and FTD, but the patient’s phenotype was not illustrated (8). The authors raised the possibility of a digenic myopathy (8), which up to date has not been proven.

Herein, we describe the clinical and pathological phenotype of three unrelated probands harboring the combined heterozygous *TIA1* and *SQSTM1* variants in the setting of MRV or myofibrillar pathology, providing evidence that co-occurrence of these variants are associated with late-onset myopathy.

## Patients and Methods

### Patients

Three patients with distal myopathy of unknown molecular defect were identified in the neuromuscular clinics during their clinical evaluation. Clinical history and findings and serological, electrophysiological, muscle pathological, and radiological data were reviewed. All patients provided written informed consent. The study was approved by the respective research ethics boards of Mayo Clinic (Institutional Review Board), Rochester, MN, USA and University of Calgary, Canada. We also obtained written and informed consent from the patients who gave specific permission to publish the data.

### Methods

#### Morphological Studies

Conventional histochemical studies were performed on fresh-frozen muscle biopsy 10-µm thick sections and stained for hematoxylin-eosin, modified Gomori trichrome, NADH dehydrogenase, succinate dehydrogenase, cytochrome c oxidase, adenosine triphosphatase (at pH 4.3, 4.6, and 9.4), acid phosphatase, periodic acid–Schiff, oil red O, non-specific esterase, and Congo red. Amyloid deposits were identified in Congo red stain sections viewed under rhodamine optics. For immunocytochemical studies cryostat sections 6–10 µm thick were reacted with monoclonal antibodies against myotilin (Novocastra, Bannockburn, IL, USA), alpha-B-crystallin (Stressgen, Ann Arbor, MI, USA), desmin (Dako, Carpinteria, CA, USA), and dystrophin (Novocastra), as previously described (9). Families 1 and 2’s proband’s muscle sections were also reacted with monoclonal antibodies against TIA1 (Abcam, Cambridge, UK) and family 3’s proband’s muscle sections against TAR DNA binding protein 43 (Proteintech, Rosemont, IL, USA). The immunoreactive sites were visualized with secondary antibodies using immunoperoxidase (9).

#### Clinical Molecular Genetic Studies

Nineteen genes known to be causative of distal myopathy (*ANO5, BAG3, CAV3, CRYAB, DES, DNAJB6, DYSF, FHL1, FLNC, GNE, LDB3, MATR3, MYH7, MYOT, SQSTM1, TCAP, TIA1, TTN* and *VCP*) were tested by next-generation sequencing (NGS) in a commercial diagnostic laboratory (PreventionGenetics, Marshfield, WI, USA) in all three probands. All coding regions plus 20 bp flanking non-coding regions of these genes were analyzed by a combined targeted NGS and Sanger sequencing, as previously described (10). In addition, *SQSTM1* aCGH microarray analysis was performed in patient 1. *ACTA1, SEPN1*, and *LMNA* were analyzed by targeted NGS in family 2’s proband because of the myofibrillar pathology.

#### Expanded NGS Panel and Whole Exome Sequencing (WES) Studies

As clinical gene analysis was thought to be non-diagnostic, additional molecular testing was performed at Mayo Clinic on genomic DNA samples. This included expanded NGS panels with a 141 gene myopathy panel and a 217 gene distal weakness panel (Data S1 in Supplementary Material) in family 2’s proband and WES in family 1’s proband. The expanded NGS panels test coding regions, splicing sites, and key regulatory regions of genes of interest using a custom reagent were developed by Mayo Clinic Genomics Laboratory and Agilent Technologies. Copy number variation (CNV) analysis was conducted based on the depth of coverage using the PatternCNV pipeline in comparison with internal reference controls (11) (and *manuscript in preparation*). Variant interpretation was conducted according to ACMG guidelines. Gene list and variants detected are included in the supplemental data (Table S1 in Supplementary Material). WES was performed using Agilent SureSelect Human All exon V5 capture reagent, and it was sequenced on Illumina HiSeq at the Medical Genome Facility at Mayo Clinic. Routinely, >95% of target region was sequenced at >20×. Sequencing results were processed using in house developed pipeline and annotated using Ingenuity (Qiagen Inc.) and Alamut Batch (Alamut) software for clinical interpretation (12, 13). DNA CNV analysis by PatternCNV and manual data review were conducted to scan for large copy number alteration (11). To identify variants of interest, we adopted an exome interpretation strategy described previously (12, 14). Briefly, after applying standard quality filter and tiered population frequency filters, variants in genes known to cause muscle diseases (based on OMIM, HGMD) were individually reviewed according to ACMG guidelines. Next, predicted high-impact variants and variants in muscle expressed genes (371 genes, accessed in December 2015, www.proteinatlas.org) (15) were examined as candidate findings. Detailed WES procedure and variant statistics are provided in supplemental data (Data S1 in Supplementary Material) due to the amount of variants detected.

#### Testing of Family Members

Targeted Sanger sequencing analyses were performed on probands and relative’s available samples at Medical Genome Facility at Mayo Clinic for families 1 and 2 and at Dr. Pfeffer’s laboratory at Hotchkiss Brain Institute for family 3.

Reference sequences were used: for TIA1, we used NM_022173.2, and for SQSTM1, we used NM_003900.4.

## Results

**Figure 1:**
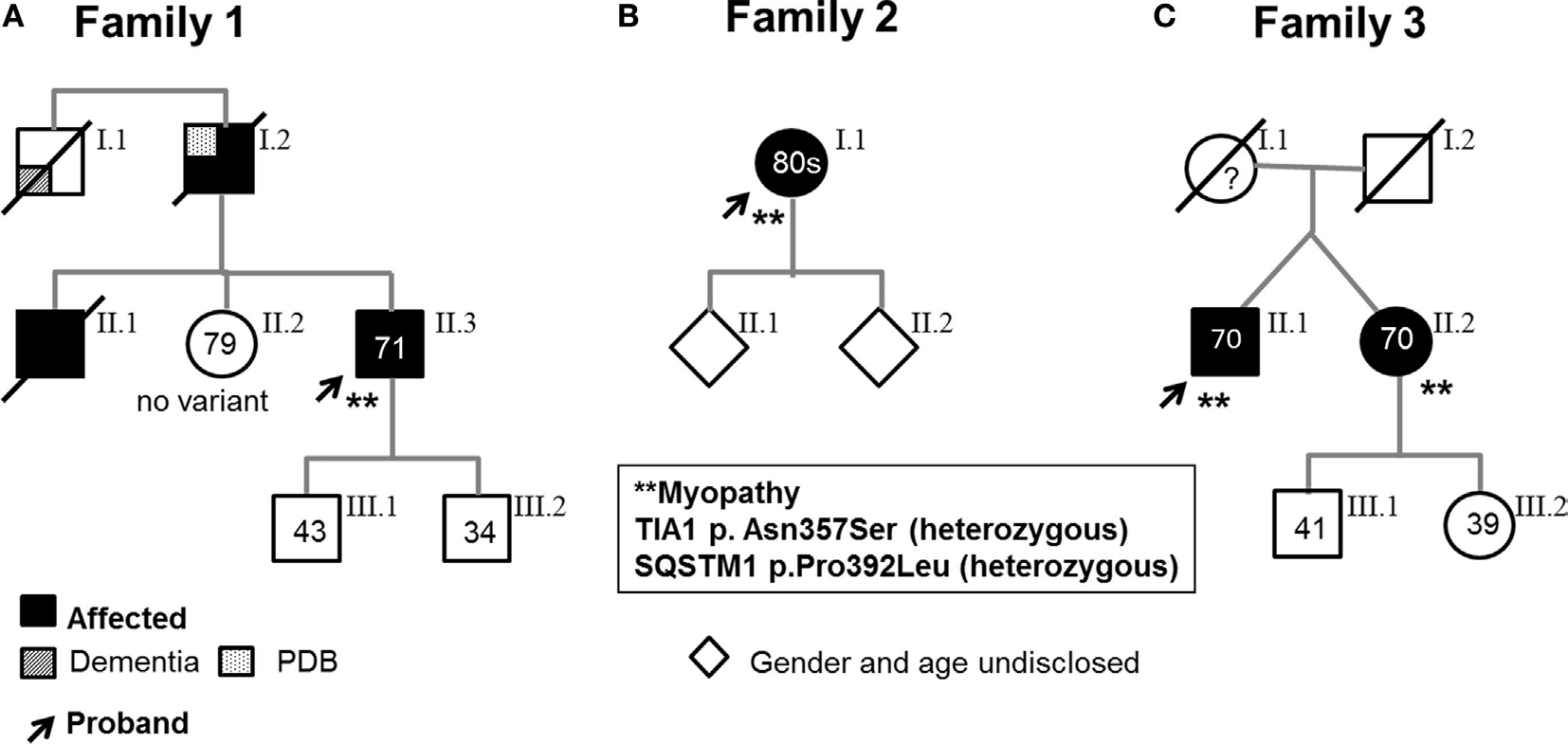
Pedigrees. Arrows indicate probands, who are heterozygous for both the *SQSTM1* and *TIA1* variants and have a muscle biopsy-proven myopathy. **(A)** Family 1. Proband 1’s asymptomatic sister (II.2) carries neither variant; the two asymptomatic sons carry neither variant nor the *SQSTM1* variant. **(B)**. Family 2. One of Proband 2’s asymptomatic children harbors the *TIA1* variant. **(C)**. Family 3. Affected dizygotic twin siblings carry both *TIA1* and *SQSTM1* variants (only the probands underwent muscle biopsy). The asymptomatic son and daughter carry neither variant nor only the *TIA1* variant. As some of the probands’ children elected not to learn about their genetic status, carriers of specific variants are not indicated. (All living affected subjects were examined; the asymptomatic individuals were examined and had no weakness or were interviewed over the phone and denied weakness and symptoms suggestive of myopathy.)

**Figure 2:**
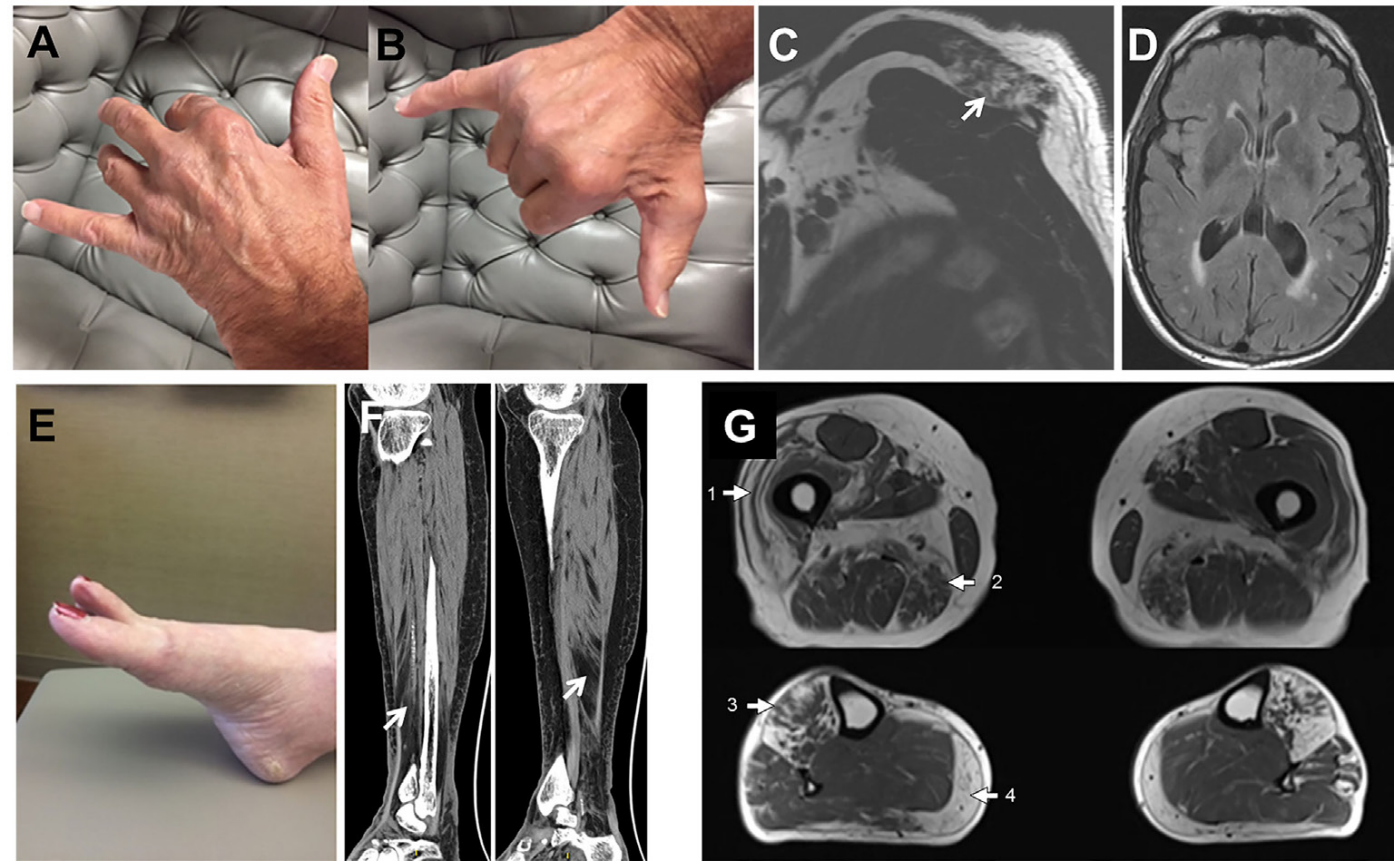
Patient’s photographs and radiological images. Proband 1 **(A–D)**. Photographs illustrating the predominant weakness of digit II to IV extensors with spared I and V digit extensors **(A,B)**. MRI images demonstrating focal fatty atrophy (arrow) involving the left posterior trapezius muscle **(C)**. T2 FLAIR brain MRI images showing mild generalized atrophy with patchy and confluent periventricular white matter hyperintensities suggestive of leukoaraiosis **(D)**. Proband 2 **(E,F)**. Photograph demonstrating the hanging big toe **(E)**. CT images revealing moderate-marked fatty atrophy involving the anterior compartment musculature of the right lower leg overlying the distal third of the tibia and fatty infiltration of the lower calf muscle **(F)**. Proband 3 **(G)**. Muscle MRI showing fatty infiltration of muscles in a geographic distribution, indicated by numbered arrows. The predominantly affected muscles include the vastus lateralis (1) and adductor magnus (2) in the thighs and anterior compartment muscles (3) and medial gastrocnemius (4) in the lower legs.

**Figure 3:**
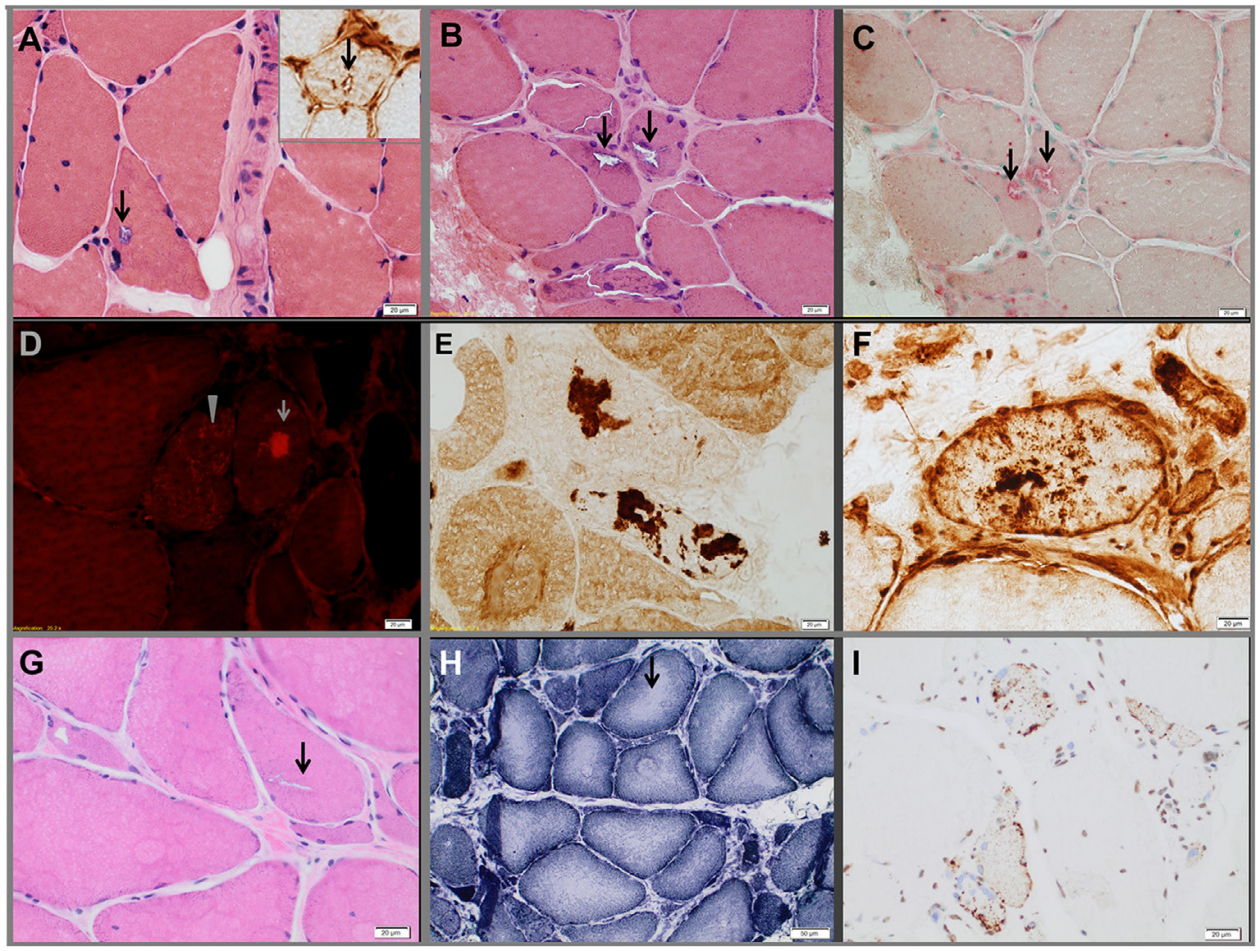
Muscle biopsies from the three probands. Proband 1 **(A)**. Hematoxylin-eosin-stained section demonstrating a vacuole (arrows) rimmed by membranous material. The insert in the upper right corner shows a TIA1-positive inclusion within a vacuole. Proband 2 **(B–F)**. Hematoxylin-eosin-stained section **(B)** showing rimmed vacuoles (arrows), which strongly overreact for acid phosphatase **(C)**. Extravacuolar large (arrow) or small congophilic inclusions (bright red dots within muscle fiber pointed by arrow head) **(D)** are present in structurally abnormal fibers (congo red stained section viewed under rhodamine optics). Several fibers demonstrated focal accumulation (dark stain) of myotilin [**(E)**, representative fiber], TIA1 [**(F)**, representative fiber], alpha-B crystallin, and desmin (data not shown). The pathological findings are suggestive of myofibrillar myopathy. Proband 3 **(G–I)**, hematoxylin-eosin-stained section showing chronic myopathic changes and rimmed vacuoles (arrow) **(G)**. NADH-TR-stained section revealing “rubbed-out” areas of decreased oxidative enzyme reactivity (arrow, representative fiber) in multiple myofibers **(H)**. Several fibers demonstrating mislocalized cytoplasmic granules of TDP-43 staining (dark stain) **(I)**.

Pedigrees are represented in Figures 1A–C; clinical and laboratory findings are summarized in the Table 1 and illustrated in Figures 2 and 3.

**Table 1:** Clinical and laboratory findings of subjects carrying both the *TIA1* and *SQSTM1* variants.

	Proband 1	Proband 2	Proband 3	Patient 3.2
Age, sex	71, M	80–85, F	70, M	70, F

Age at onset (years)	65	70–75	55	45

First symptom	Index extensor weakness	Dyspnea	Finger extensors weakness	Finger extensor weakness

Weakness, UL^a^	Finger extensors > > wrist extensors	Finger extensors > > wrist extensors	Finger extensors > > wrist extensors	Finger extensors > > wrist extensors
	
	Shoulder girdle muscles (mild)	Shoulder girdle muscles (mild)	Arm muscles (mild)	Arm muscles (mild)

Weakness, LL^a^	Toe extensors > ankle dorsiflexors	Toe extensors > ankle dorsiflexors	Toe extensors > ankle dorsiflexors	Left foot drop
	
	Pelvic girdle (mild)	Pelvic girdle (mild)	Pelvic girdle (mild)	Pelvic girdle (mild)

Gait	High stepping	High stepping	High stepping	High stepping

Ability to walk on toes	No (spared toe flexors)	Mildly impaired	Mildly impaired	Mildly impaired

Tendon reflexes	Absent at ankles	Absent at ankles	Normal	NA

CK	352–784 U/L (nl < 336)	220–331 U/L (nl < 222)	Normal	NA

Cardiac involvement^b^	None	Bradycardia, atrial fibrillation, pacemaker	None	NA

Respiratory involvement	None	↓Max exp press, nocturnal O_2_ desaturation	None	NA

EMG findings	Myopathic; mild neurogenic changes distally	Myopathic	Myopathic	NA

Muscle imaging	Atrophy trapezius (MRI)^c^	Atrophy anterior compartment; fatty infiltration lower calf muscles (CT)	Fatty infiltration adductor magnus, vastus medialis, anterior compartment, and medial gastrocnemius (MRI)	NA

PBD^d^	None	None	NA	NA

MRI brain	Mild generalized atrophy	NA	Normal	NA

Cognitive assessment	Mild impaired verbal learning/retention, semantic fluency	Normal	Normal	Normal

*^a^The weakness was asymmetric in probands 1 and 2 and patient 3.2*.

*^b^Cardiac involvement was assessed by EKG and echocardiogram*.

*^c^No dedicated muscle imaging studies were performed in proband 1, but a shoulder MRI showed focal fatty atrophy of the posterior aspect of the left trapezius*.

*^d^PDB was assessed by measurement of alkaline phosphatase, including bone isoenzyme, and skeleton radiographs*.

*CK, creatine kinase; EMG, electromyography; LL, lower limbs; NA, not assessed; PDB, Paget disease of bone; UL, upper limbs*.

### Family 1

The proband is a 71-year-old man (Figure 1A) who noted difficulty extending his index finger, followed by more diffuse asymmetric distal weakness and atrophy in the upper limbs and, a year later, in the lower limbs. Recently, he has shown short-term memory deficits, difficulty findings words and organizing thoughts, and irritability. His neurological examination showed a predominant distal weakness mainly affecting finger extensors (Figures 2A,B) ankle dorsiflexors and toe extensors (Table 1). Toe flexors were spared. Creatine kinase (CK) value was mildly elevated (Table 1). Alkaline phosphatase was normal twice in the past 4 years, including bone isoenzyme. Needle electromyography (EMG) studies showed myopathic changes especially in the distal muscles with few fibrillation potentials and some neurogenic motor unit potentials in few muscles. Nerve conduction studies were normal. Shoulder MRI, performed to investigate joint pain, incidentally revealed focal fatty atrophy of the trapezius (Figure 2C). Deltoid biopsy showed an MRV (Figure 3A). Scattered vacuoles overreacted for TIA1. LAMP2 immunoreactivity was normal (data not shown). Neuropsychological assessment showed mildly impaired verbal learning/retention and semantic fluency, while brain MRI revealed mild generalized atrophy and leukoaraiosis (Figure 2D). There was no evidence of cardiac involvement by EKG and echocardiogram. Radiographs of axial and appendicular skeleton showed no features of PDB. His father, of Swiss descent, had PDB and manifested progressive distal weakness in his 70s. He was unable to extend his fingers and reportedly used his thumbs to hold a cup. He developed foot drop and lost ambulation in his early 80s. Proband’s sister and children are asymptomatic, while a brother, who died of cancer, had hand weakness in the late 50s (Figure 1A).

### Family 2

The proband is an 80- to 85-year-old woman with progressive exertional dyspnea, followed by dysphagia, progressive distal limb weakness mainly involving ankle dorsiflexors, and myalgia. She had a pacemaker placed at the age of 65 years for bradycardia and atrial fibrillation and history of gastric bypass. She had no family history of weakness, PDB, or dementia. Her examination revealed predominantly distal asymmetric weakness (Figure 2E and Table 1). CK value was slightly elevated in one occasion (231 U/L; normal < 222 U/L) and normal in another occasion. Alkaline phosphatase was normal twice, including bone isoenzyme. EMG study showed myopathic changes especially in the weakest muscles and diaphragm with fibrillation potentials in the tibialis anterior. Nerve conduction studies and 2 Hz repetitive nerve stimulations of the ulnar and spinal accessory nerves were normal. CT of the leg showed distal fatty atrophy of the muscles (Figure 2F). Pulmonary function tests were significant for reduced maximal expiratory pressure to 63% of predicted, while overnight oximetry showed desaturation to 80% (patient declined nocturnal polysomnography). Tibialis anterior biopsy (Figures 3B–F) showed features of myofibrillar myopathy (16). In ATPase reacted sections, there was grouping of type 1 fiber in several fascicles suggesting reinnervation (data not shown). Proband 2 was patient #9 in a case series of myopathy with respiratory insufficiency (17). No bone abnormalities were evident by leg CT or hip radiograph. EKG revealed a controlled ventricular rate in the setting of electronic atrial pacemaker; 24-h-Holter monitoring and echocardiogram were unrevealing.

### Family 3, Proband (Patient II.1)

The proband is a 70-year-old man of British descent (Figure 1C) who developed difficulty fully extending his index finger bilaterally at the age of 55 years. A few years later, he noted that he would catch his toes on the curb while walking and had a high-stepping gait. He was found to have mild finger extensor and ankle dorsiflexor weakness. A muscle biopsy showed MRV (Figures 3G–I). His weakness did not significantly progress; he remained ambulant. At the age of 70 years, he has bilateral distal atrophy of intrinsic hand and foot muscles and mild left scapular winging (Table 1). The weakness affected preferentially distal extensor muscles. CK level was normal. EMG study showed myopathic changes. Muscle MRI showed fatty infiltration predominantly of adductor magnus and medial vastus lateralis in the thighs, anterior compartment muscles, and medial gastrocnemius in the legs (Figure 2G). Patient had no evidence of cardiac dysfunction by EKG and echocardiogram. His mother had difficulty with stairs around age 60 but did not seek medical attention and remained ambulant until death at age 89. His sister (Patient 3.2 below) has weakness. No other family members have any neurological symptoms.

### Family 3, Patient II.2

She is a 70-year-old woman and fraternal twin of proband II.1 (Figure 1C). She developed weakness of right index finger extension in her mid 40’s, followed by left foot drop in her mid 50’s. At the age of 60 years, she noted slowly progressive left finger extensor weakness and cannot extend her fingers against gravity at 70 years of age. Recently, she has developed right finger extensor weakness (Table 1). She has two children and two grandchildren with no neurological symptoms.

### Molecular Data

All three probands carry two heterozygous variants: *SQSTM1*, c.1175C>T (p.Pro392Leu), and *TIA1*, c.1070A>G (p.Asn357Ser). None of the unaffected family members harbor both variants (Figure 1). The *TIA1* variant and *SQSTM1* variants have been reported in multiple databases. The *SQSTM1* variant is designated as rs104893941 in dbSNP and reported at allele frequencies of 0.0009 in the Exome Aggregation Consortium database (ExAC) (18), 0.0024 in 1,000 Genomes Project database (TGP) (19), and 0.0015 in the NHLBI GO Exome Sequencing Project (GO-ESP) (accessed January 23, 2018) (19, 20) and is more frequent in certain European populations. The *TIA1* variant is designated as rs116621885 and reported at allele frequencies of 0.0071 in ExAC, 0.0016 in TGP, and 0.0068 in GO-ESP (accessed January 23, 2018). We examined the genotype data in the TGP to determine whether these variants coincide in controls (21). None of 2,504 self-declared healthy individuals in TGP has both *TIA1*, c.1070A > G (p.Asn357Ser) and *SQSTM1*, c.1175C > T (p.Pro392Leu). No other pathogenic or suspected pathogenic variants in genes associated with muscle diseases were identified in the proband of family 2 by expanded NGS panel studies or in the proband of family 1 by WES analysis.

We are aware of a prior study in which this *SQSTM1* mutation may be part of a common founder haplotype including the following four loci: [Chr5: 179260153C/T, refSNP ID rs4935; Chr5: 179260213G/A, rs4797; Chr5: 179264731T/C, rs10277; Ch5: 179264915G/T, rs1065154 (22)]. On the basis of our available sequencing data, we attempted to infer the haplotype of the *SQSTM1* mutation for each family. The proband from Family 1 is consistent with the H1 haplotype based on the presence of homozygous genotypes for rs4935 and rs4797 although this is not definitive because the rs10277 and rs1065154 polymorphisms were not covered. The haplotype of the proband from Family 2 could not be determined based on the available genotype data. For Family 3, sequencing data were available for four family members, and we manually reconstructed the haplotype assuming the minimal number of recombinations. The result indicated that Family 3’s haplotype was consistent with either the H2 or the H5 haplotype described in the study by Lucas et al. (22). On the basis of these results, our three families have at least two different haplotypes associated with the *SQSTM1* mutation, indicating that this unique phenotype is not a haplotype-specific effect, as well as demonstrating that these families are not remotely related to each other.

## Discussion

We present the first detailed clinical and pathologic data from three unrelated families with predominant distal myopathy associated with a known pathologic variant in *SQSTM1* (p.Pro392Leu) and a variant in *TIA1* (p.Asn357Ser). At the time of this report, only a single prior myopathy case with the same genetic variants has been reported, but the clinical and myopathological features were not illustrated (8). There are also two further cases of MRV having the same *TIA1* variant but a different *SQSTM1* mutation (c.1165+1G>A) (8), one of whom was previously reported as having a SQSTM1-MRV (6). Although the causality of the coexisting *SQSTM1* and *TIA1* variants in myopathy has not been proven, our affected individuals from three unrelated family provide further support to the digenic nature of this myopathy. In addition, we have identified a novel genetic defect (the combined *SQSTM1* and *TIA1* variants) associated with myofibrillar pathology, which to our knowledge has not been previously described in association with *TIA1* and *SQSTM1* variants, in isolation or in combination.

The contributing role of the two detected variants in causing the myopathy is supported by the following: (1) the *SQSTM1* p.Pro392Leu mutation is a known pathogenic variant previously associated with ALS, FTD, and/or PDB, and although by itself it has never been reported in association with a myopathy, a different *SQSTM1* mutation was detected in an MRV case (6); (2) the *TIA1* variant p.Asn357Ser (designated as rs116621885 in dbSNP) is less likely to be causative by itself of a dominant disease, given the population frequency of 0.71% in ExAC databases and has never been shown to segregate with disease, except in combination with *SQSTM1* mutations (8, 18); (3) all our four patients from three independent families with myopathy have both genetic variants, and the likelihood that these two variants occurring in three families by chance (given they are on different chromosomes) is extremely small; (4) co-segregation of these two variants is not detected in the self-declared healthy subjects of the TGP database (21); (5) exome sequencing in proband 1 and a 362-gene neuromuscular disorders panel in proband 2 did not identify an alternative genetic etiology; and (6) there is plausible biological rationale for an interaction between variants in *TIA1* and *SQSTM1* and their interplay in causing myopathy because both genes are involved in protein quality control *via* stress granule assembly and autophagy, respectively. SQSTM1 is localized adjacent to stress granules for extraction of ubiquitinated proteins for autophagy (6), suggesting the possibility that genetic variations in both *TIA1* and *SQSTM1* can interact and disrupt this pathway. However, molecular and functional studies to dissect the likely interaction between TIA1 and SQSTM1 would shed light on the pathomechanism of the myopathy. Given that the *TIA1* p.Asn357Ser variant is relatively common, and therefore likely not pathogenic by itself, we speculate that the *TIA1* variant may have a modifier effect on the phenotype related to the pathogenic *SQSTM1* mutations. The digenic nature of the myopathy is now supported by the presence of myopathy in five unrelated probands [three from this report and two from the study by Evila et al. (8)], all carrying a pathogenic *SQSTM1* mutation and the *TIA1* p.Asn357Ser variant.

The patients reported here show phenotypic variability, a well-recognized feature of late-onset hereditary myopathies, although three of them manifested with index extensor weakness, a classic feature of *TIA1* myopathy. The myopathic respiratory insufficiency and atrial fibrillation (although the latter finding could an incidental finding) in family 2’s proband emphasize the need to investigate respiratory and cardiac function in future cases of *SQSTM1*/*TIA1* myopathy.

Myofibrillar myopathy, detected in family 2’s proband, has not been previously described in association with *TIA1* or *SQSTM1* variants, in isolation or in combination. Such finding is not surprising because of the known pathological overlap between MRV and myofibrillar myopathy. However, in light of our observation, we suggest that *SQSTM1/TIA* analysis is included in the molecular investigations of myofibrillar myopathies.

The cognitive impairment in family 1 may reflect the previously reported deleterious effects of the *TIA1* and *SQSTM1* variants on brain, as mutations in both genes have been identified in ALS and FTD (1). In addition, one could speculate that the histopathological features of reinnervation observed in proband 2’s muscle biopsy might signal a coexisting neurogenic component due to the underlying genetic makeup.

Large genomic sequencing projects have enabled studies on the causes of complex genetic disease and often broad clinical manifestations (18, 21). Recent studies have highlighted that digenic interactions in genetic diseases, especially in neuromuscular disorders, may be more frequent than previously appreciated (23–25). There is indeed emerging evidence that many genetic factors can contribute to the phenotype variability, especially in complex neuromuscular disease (25–27). The cases described here support the importance of considering the interaction among multiple genetic variants in determining the patient’s phenotype, disease mechanisms, and modality of inheritance. The lack of functional studies is certainly a limiting feature of the study, and further investigations will be needed to prove the digenic nature of the myopathy and the deleterious effects of the combined *SQSTM1* and *TIA1* variants in myopathy.

## Ethics Statement

All patients provided written informed consent. The study was approved by the respective research ethics boards of Mayo Clinic (Institutional Review Board), Rochester MN, USA and University of Calgary, AB, Canada. We also obtained written and informed consent from the patients who gave specific permission to publish the data.

## Author Contributions

Study concept and design: MM, ZN, and GP. Acquisition, analysis, or interpretation of data: all authors. Drafting of the manuscript: ZN, GP, and MM. Critical revision of the manuscript for important intellectual content: ZN, AE, LH, EN, GP, MM. Obtained funding: MM. ZN and CP contributed equally. GP and MM contributed equally.

## Conflict of Interest Statement

Author AG was employed by company Prevention Genetics. All other authors declare no competing interests.
