# Supplementary material for: Myopathy With SQSTM1 and TIA1 Variants: Clinical and Pathological Features
Source: Front Neurol. 2018 Mar 19;9:147. doi: 10.3389/fneur.2018.00147 (PMC5868303; doi:10.3389/fneur.2018.00147)
Supplement: Supplementary file 1 [file data_sheet_1.DOCX]

**Supplemental Data, S1.**

**Expanded NGS panel gene lists.**

**1)** **NMPAN- Myopathy Expanded panel:** *ABHD5, ACAD9, ACADL, ACADM, ACADS, ACADVL, ACTA1, ADGRG6, AGL, ANO5, B3GALNT2, B4GAT1, BAG3, BIN1, BVES, CAPN3, CAV3, CAVIN1, CCDC78, CFL2, CHKB, CNTN1, COL12A1, COL6A1, COL6A2, COL6A3, COQ2, COQ4, COQ6, COQ8A, COQ9, CPT1B, CPT2, CRYAB, DAG1, DES, DMD, DNAJB6, DNM2, DOLK, DPM1, DPM2, DPM3, DYSF, EMD, ENO3, ETFA, ETFB, ETFDH, FAM111B, FHL1, FKRP, FKTN, FLNC, GBE1, GGPS1, GMPPA, GMPPB, GNE, GOSR2, GYG1, GYS1, HADHA, HADHB, HNRNPA1, HNRNPA2B1, HNRNPDL, HRAS, ISPD, ITGA7, KBTBD13, KLHL40, KLHL41, KY, LAMA2, LAMP2, LARGE, LDB3, LDHA, LMNA, LPIN1, LRSAM1, MATR3, MTM1, MYF6, MYH2, MYH7, MYO18B, MYOT, NEB, NHLRC1, ORAI1, PDSS1, PDSS2, PFKM, PGAM2, PGK1, PGM1, PHKA1, PLEC, PNPLA2, POMGNT1, POMGNT2, POMK, POMT1, POMT2, PRKAG2, PYGM, RBCK1, RYR1, SBDS, SELENON, SGCA, SGCB, SGCD, SGCG, SLC22A5, SLC25A20, SMCHD1, SPEG, SQSTM1, SRPK3, STAC3, STIM1, SYNE1, TCAP, TIA1, TMEM43, TMEM5, TNNT1, TNPO3, TPM2, TPM3, TRAPPC11, TRDN, TRIM32, TRIM54, TRIM63, TTN, VCP, VMA21*.

**2)** **NMPAN- Distal Weakness Expanded Panel:** *AAAS, AARS, ABCA1, ABCD1, ACTA1, ADCY6, AIFM1, AMACR, ANO5, AP1S1, AP4B1, AP4E1, AP4M1, AP4S1, AP5Z1, APOA1, APTX, ARHGEF10, ARSA, ATL1, ATM, ATP7A, B2M, B4GALNT1, BAG3, BCKDHB, BICD2, BIN1, BSCL2, C12orf65, CAV3, CCT5, CLCF1, CNTNAP1, COX10, CPOX, CRLF1, CRYAB, CTDP1, CTSA, CYP27A1, CYP2U1, CYP7B1, DARS2, DCAF8, DCTN1, DDHD1, DDHD2, DES, DGUOK, DHH, DHTKD1, DNAJB2, DNAJB6, DNM2, DNMT1, DST, DYNC1H1, DYSF, EGR2, ERBB3, ERCC6, ERCC8, ERLIN2, FA2H, FAH, FAM126A, FAM134B, FBLN5, FBXO38, FGD4, FGF14, FHL1, FIG4, FLNC, FLVCR1, FMR1, GALC, GAN, GARS, GBA2, GBE1, GDAP1, GJB1, GJB3, GJC2, GLA, GNB4, GNE, GSN, HADHA, HADHB, HARS, HINT1, HK1, HMBS, HNRNPA1, HNRNPA2B1, HSPB1, HSPB3, HSPB8, HSPD1, IGHMBP2, IKBKAP, INF2, KARS, KIF1A, KIF1B, KIF5A, L1CAM, LAMA2, LDB3, LITAF, LMNA, LRSAM1, LYST, MAF, MARS, MATR3, MED25, MFN2, MMACHC, MPV17, MPZ, MTMR2, MTTP, MYH14, MYH2, MYH7, MYOT, NAGA, NAGLU, NDRG1, NEB, NEFL, NF2, NGF, NHLRC1, NIPA1, OAT, OPA1, PANK2, PDHA1, PDK3, PDYN, PEX10, PEX7, PHYH, PLA2G6, PLEKHG5, PLOD1, PLP1, PMM2, PMP2, PMP22, PNKP, PNPLA6, POLG, PPOX, PRNP, PRPS1, PRX, RAB7A, REEP1, RRM2B, RTN2, SACS, SBF1, SBF2, SCN10A, SCN11A, SCN9A, SCO2, SCP2, SELENON, SETX, SH3TC2, SLC12A6, SLC16A2, SLC25A19, SLC25A46, SLC33A1, SLC52A2, SLC5A7, SNAP29, SOD1, SOX10, SPAST, SPG11, SPG20, SPG21, SPG7, SPTLC1, SPTLC2, SQSTM1, SURF1, TDP1, TECPR2, TFG, TIA1, TRIM2, TRPA1, TRPV4, TTN, TTPA, TTR, TUBB3, TWNK, TYMP, VCP, VPS37A, WASHC5, WNK1, XPA, XPC, YARS, ZFYVE26*.

**Molecular Genetics analysis.**

**Expanded NGS panel testing of the proband of family 2**: As clinical gene analysis was initially thought to be non-diagnostic, a 217 gene Distal Weakness Expanded Panel and a 141 gene Myopathy Expanded Panel were performed on the proband of family 2 (see gene list below). All coding regions and key regulatory regions of these genes were analyzed by targeted NGS next generation sequencing. The proband’s genomic DNA was sheared by sonication, captured using a custom reagent developed by Mayo Clinic Genomics Laboratory and Agilent Technologies, and sequenced (as 2x101 bp) on Illumina Hiseq platform with an average coverage of ~1800X on target region. Sequencing data analysis was performed using CLC biosciences and in house developed NGS pipelines. Copy number analysis on the coding regions of genes of interest was conducted based on depth of coverage using the PatternCNV pipeline in comparison with internal reference controls (11, and manuscript in preparation). Regions with coverage lower than 100X were subjected to raw data manual inspection. Genetic variants identified were annotated using in house developed annotation pipeline and was interpreted according to ACMG guidelines (Supplemental Table 1).

**WES analysis of the proband of family 1** WES was performed on genomic DNA from the proband of family 1. Exome was captured using Agilent SureSelect Human All exon V5 capture reagent and sequenced on Illumina HiSeq NGS platform at the Medical Genome Facility of Mayo Clinic. Routinely, >95% of the target regions were sequenced at >20X. Raw sequencing data was aligned to human reference genome (hg19) using Novoalign (Novocraft Technologies), genomic variants were jointly called using GATK Haplotype Caller and GenotypeGVCF (13) and annotated using Ingenuity (Qiagen Inc), Alamut Batch (Alamut) software. Total of ~91k variants were detected in this sample. To identify variants of interest, we adopted an exome interpretation strategy described previously (14, 12). Briefly, two population minor allele frequency filters based on ExAC, ESP, and thousand genome project, were applied as following: 1) 2.0% on variants which are reported previously in Human Gene Mutation Databases or ClinVar, 2) 1.0% on variants not previously described in either databases. Total of ~8400 variants passed standard quality filter and population frequency filters. Next variants in genes associated with known muscle diseases were evaluated according to ACMG guidelines based on disease inheritance pattern, frequency, functional evidence, molecular impact, and prediction score. Subsequently variants in muscle expressed genes (371 genes accessed in Dec 2015 from www.proteinatlas.org, 15) were examined based on their impact on gene and protein function. As these genes have not been associated with human disease or phenotype, future studies are needed to clarify the effects. PatternCNV analysis and manual data check were performed to exclude large copy number alterations only due to the limitation of WES coverage.

**Supplemental Table 1. Result of expanded NGS panel testing of the proband of family 2**

| Chr | Position | Gene | transcript | Alteration | Zygosity | OMIM-Phenotype (inheritance) | Classification |
| --- | --- | --- | --- | --- | --- | --- | --- |
| 5 | 179263445 | *SQSTM1* | NM_003900.4 | c.1175C>T  p.Pro392Leu | het | Myopathy, distal, with rimmed vacuoles (AD); Paget disease of bone 3 (AD); Frontotemporal dementia and/or amyotrophic lateral sclerosis 3 (AD); Neurodegeneration with ataxia, dystonia, and gaze palsy, childhood-onset (AR) | Pathogenic |
| 2 | 70439942 | *TIA1* | NM_022173.3 | c.1070A>G  p.Asn357Ser | het | Welander distal myopathy (AD/AR) | VUS |
| 1 | 235667522 | *B3GALNT2* | NM_152490.4 | c.31T>G  p.Cys11Gly | het | Muscular dystrophy-dystroglycanopathy (congenital with brain and eye anomalies, type A, 11) (AR) | VUS |
| 11 | 68707054 | *IGHMBP2* | NM_002180.2 | c.2837G>A  p.Arg946Gln | het | Neuronopathy, distal hereditary motor, type VI, AR; Charcot-Marie-Tooth disease, axonal, type 2S, AR; | VUS |
| 15 | 44877834 | *SPG11* | NM_025137.3 | c.5121G>T  p.Glu1707Asp | het | Spastic paraplegia 11 (AR); Charcot-Marie-Tooth disease, axonal, type 2X (AR); Amyotrophic lateral sclerosis 5, juvenile (AR) | VUS |
| 17 | 40690442 | *NAGLU^*^* | NM_000263.3 | c.617A>G  p.Asn206Ser | het | ?Charcot-Marie-Tooth disease, axonal, type 2V (AD); Mucopolysaccharidosis type IIIB (Sanfilippo B) (AR) | VUS |
| 17 | 40847566 | *CNTNAP1* | NM_003632.2 | c.3020C>G  p.Thr1007Ser | het | Lethal congenital contracture syndrome 7 (AR) | VUS |
| 19 | 50334047 | *MED25* | NM_030973.3 | c.1004C>T  p.Ala335Val | het | Basel-Vanagait-Smirin-Yosef syndrome (AR); ?Charcot-Marie-Tooth disease, type 2B2 (AR) | VUS |
| 4 | 100518330 | *MTTP* | NM_001300785.1 | c.1097C>A  p.Ala366Glu | het | Abetalipoproteinemia, 200100 (AR) | VUS |

Abbreviations: AD= autosomal dominant; AR= autosomal recessive; Chr= chromosome number; het= heterozygous; VUS= variant of unknown significance

* A single family with a mutation in *NAGLU* has been reported with autosomal dominant disease (axonal Charcot Marie Tooth disease type 2V)
